# Delayed right ventricular defibrillation lead perforation presenting as cardiac tamponade and treated surgically

**DOI:** 10.1002/ccr3.865

**Published:** 2017-03-02

**Authors:** Masahiko Noguchi, Toshiko Nakai, Yuji Kawano, Kentaro Shibayama, Kotaro Obunai, Minoru Tabata, Hiroyuki Watanabe

**Affiliations:** ^1^ Department of CardiologyTokyo Bay Urayasu Ichikawa Medical CenterChibaJapan; ^2^ Division of Advanced Therapeutics for Cardiac ArrhythmiasDepartment of MedicineNihon University School of MedicineTokyoJapan; ^3^ Department of Cardiovascular SurgeryTokyo Bay Urayasu Ichikawa Medical CenterChibaJapan

**Keywords:** Defibrillator lead, delayed perforation, pericardial effusion

## Abstract

Right ventricular perforation leading to cardiac tamponade can occur during the chronic phase after cardiac device implantation. Physicians who manage the pacemaker clinic must be alert to the wide range of symptoms and signs that can accompany delayed right ventricular perforation. Surgical rather than percutaneous lead extraction may be prudent.

## Introduction

Implantable cardioverter defibrillator lead perforation of the right ventricle is a well‐known complication, occurring most often during or soon after the implantation 1. Delayed perforation is, on the contrary, quite rare, and various time frames, symptoms, and clinical findings are reported 2. Appropriate management of such delayed perforation has, to date, not been thoroughly explored in the literature. Once delayed perforation is discovered, a decision must be made to extract the perforating lead either percutaneously or surgically, depending on the case particulars. We report a case in which cardiac tamponade resulted from perforation of the right ventricular wall that occurred during the chronic period after cardiac resynchronization therapy defibrillator implantation and for which we chose to extract the lead surgically.

## Case Report

**Figure 1:**
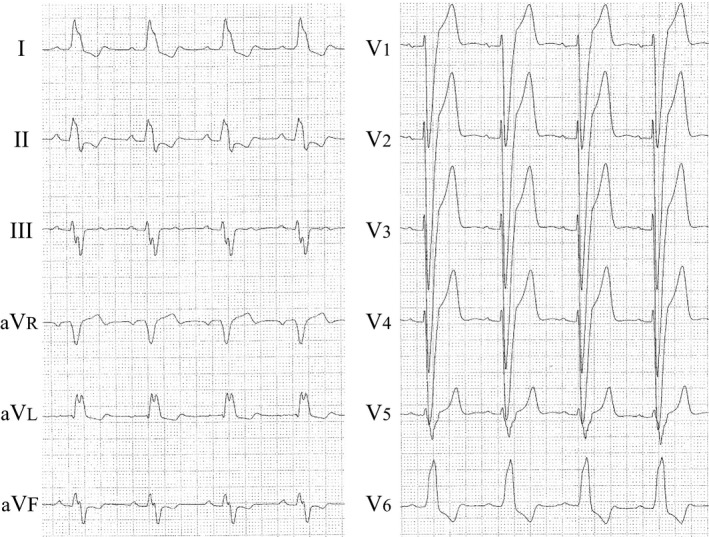
Electrocardiogram showing complete left bundle branch block with a QRS width of 200 msec.

**Figure 2:**
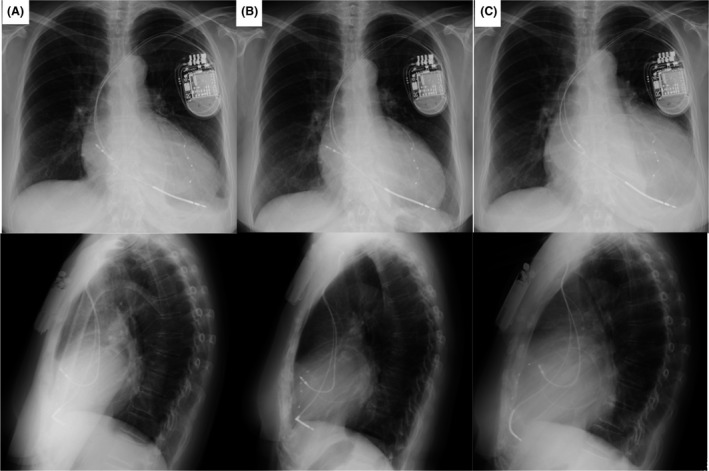
Serial posterior–anterior (upper row) and left lateral chest (bottom row) X‐ray films showing the ICD lead position. (A) Films obtained at the time of the patient's initial discharge. (B) Films obtained at the time of the first outpatient visit after implantation. (C) Films obtained at the time of re‐admission. At this time, the cardiothoracic ratio had clearly increased.

A 62‐year‐old woman with dilated cardiomyopathy was referred to our hospital with acute congestive heart failure. Despite appropriate medical therapy, she remained in New York Heart Association (NYHA) class III. Electrocardiography revealed complete left bundle branch block with a QRS width of 200 msec (Fig. 1). Echocardiography showed poor left ventricular function (ejection fraction: 20%) with intraventricular dyssynchrony. We implanted a cardiac resynchronization therapy defibrillator (CRT‐D) (Quadra Assura MP CRT‐D, St. Jude Medical, St. Paul, MN) with a transvenous active fixation implantable cardioverter defibrillator lead (Durata, St. Jude Medical). Leads were inserted via the left subclavian vein. The defibrillation lead was implanted in the right ventricular apex (Fig. 2A). After the procedure, all lead measurements were within normal range. One week after the procedure, we noted at the time that the R‐wave amplitude had decreased to 3.3 mV. Echocardiography confirmed absence of pericardial effusion, a complication that can result from lead perforation, and the patient was discharged.

**Figure 3:**
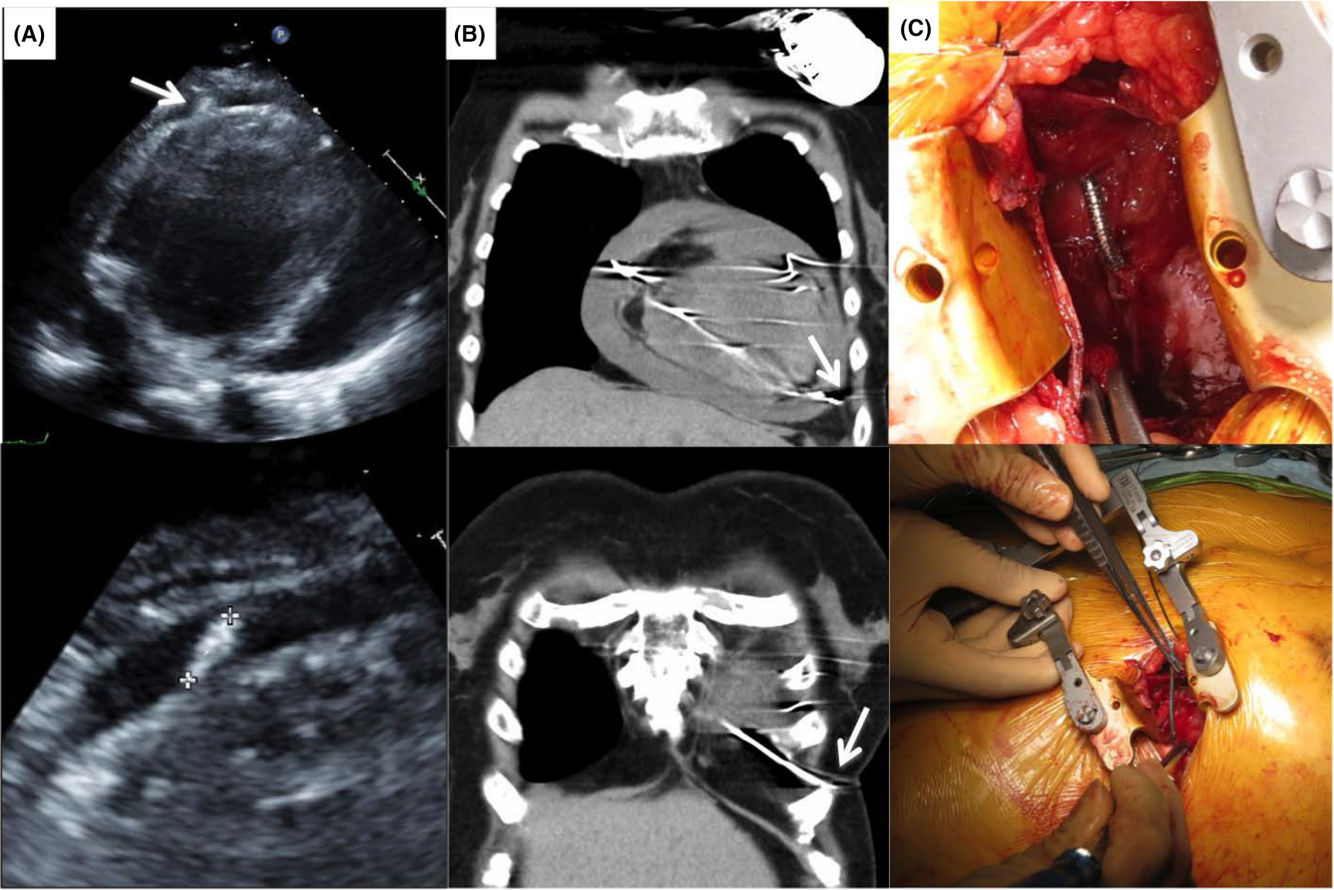
Investigation and confirmation of the cause of the patient's sudden complaints. (A) Echocardiogram showing severe pericardial effusion and the ICD lead (white arrow) extending beyond the ventricular apex. (B) Chest computed tomography image showing that the tip of the ICD lead (white arrow) had perforated the right ventricular wall and migrated beyond the epicardium. (C) Intraoperative photograph. Visual inspection confirmed that the ICD lead had perforated the right ventricular wall.

The patient visited our pacemaker clinic 3 weeks after the CRT‐D implantation, and her heart failure had improved to NYHA class I. The cardiothoracic ratio measured on a chest X‐ray film had decreased slightly, and the cardioverter defibrillator lead was in its proper place (Fig. 2B). Echocardiography was again performed, and no pericardial effusion was detected. At 51 days after device implantation, however, the patient returned to our hospital complaining of nausea, loss of appetite, and breathlessness, all of which had come on suddenly. Her blood pressure, which was usually 120/70 mmHg, had decreased to 103/53 mmHg. A blood test revealed a hemoglobin concentration of 7.9 g/dL and hematocrit of 24.4% (down from 10.6 g/dL and 31.6%, respectively, at the time of discharge). Chest X‐ray showed that the cardiothoracic ratio had increased from 59% to 71% (Fig. 2C). Echocardiography revealed a large pericardial effusion and protrusion of the defibrillation lead into the pericardial space (Fig. 3A). Chest computed tomography (CT) was also performed, and it showed that the tip of the defibrillation lead had perforated the right ventricular wall and penetrated the epicardium (Fig. 3B). CRT‐D interrogation revealed no ventricular arrhythmia, but the sensed R‐wave amplitude had dropped to 0.2 mV. Cardiac tamponade seen on echocardiography, the severe anemia, and the decreased blood pressure led to a decision to remove the defibrillation lead surgically under fluoroscopic and transesophageal echocardiographic guidance. Intercostal thoracotomy was performed, and 450 mL of bloody pericardial fluid was drained from the pericardial space. We confirmed that the defibrillation lead had perforated the right ventricular wall (Fig. 3C), and we wondered whether the marked decrease in R‐wave amplitude at the time of the patient's discharge was an indication that some micro‐dislodgment had already occurred. We disconnected the defibrillation lead from the CRT‐D generator and extracted it. A new defibrillation lead was implanted in the ventricular apex near the interventricular septum under fluoroscopic and transesophageal echocardiography guidance. We encountered no complications. The patient was discharged 4 weeks after the procedure. One year and 3 months has passed since discovery of the perforation, and she continues to do well.

## Discussion

Right ventricular perforation by a defibrillator lead is a rare but serious complication that occurs in 0.6–5.2% of patients who receive an implantable cardioverter defibrillator (ICD) 1. Acute perforation occurs during or soon after the implantation and results in hemodynamic compromise presenting as cardiac tamponade. Delayed perforation, that is, perforation occurring more than 1 month after implantation, is associated with various symptoms, and some asymptomatic cases have been reported 2. Because of the low incidence of delayed right ventricular perforation, the clinical presentation and optimal management have not been clearly defined.

In the case described herein, we learned two important lessons. The first is that we should be aware that delayed right ventricular perforation can result in sudden‐onset hemodynamic compromise indicative of cardiac tamponade in the chronic phase after device implantation. We do not expect acute perforation to be asymptomatic, and although delayed perforation is often asymptomatic, it can produce overt manifestations of cardiac tamponade. According to one report, as many as 15% of patients with CT‐confirmed delayed perforation are asymptomatic 2. In our case, 3 weeks after the CRT‐D implantation, chest X‐ray performed in the outpatient clinic showed no lead dislodgement. Careful follow‐up for up to 3 months is necessary because of the possibility of delayed right ventricular perforation in the chronic phase after device implantation. Our patient's activity level had increased dramatically to the point that she was using her bicycle regularly, and her heart had decreased somewhat in size. We believe these changes increased the risk of lead perforation. Interestingly, a group of patients reported recently all showed altered electrical measurements upon device interrogation 3. Therefore, use of a home‐monitoring system might be effective for early detection of the lead malfunction characteristic of lead perforation, whether it be a decrease in R‐wave amplitude, a decrease in pacing impedance, a loss of capture at maximal pacing output, or an increase in the ventricular pacing threshold 4. The causes of lead perforation vary, from individual patient characteristics to tip position, to lead type 3, to concomitant therapy 5. The perforation in our case might have derived from the patient's dilated cardiomyopathy, which causes thinning of the right ventricular wall. The data are insufficient regarding the safety of positioning the defibrillator lead in the right ventricular septum versus the right ventricular apex. We also think it is possible that use of an anticoagulant for prevention of left ventricular thrombosis contributed to our patient's cardiac tamponade once the perforation occurred.

The second lesson is that we should carefully decide to extract perforating leads either percutaneously or surgically. Laborderie et al. reported that leads can safely be extracted under fluoroscopic guidance with close monitoring and backup surgical support in most patients with subacute or delayed right ventricular perforation 6. However, the risk of bleeding increases rapidly in instances of delayed perforation 7. If the tip of the lead has migrated beyond the epicardium, surgical extraction is safer than percutaneous extraction because the surgical procedure allows us to visualize and repair the perforation defect and any other injuries. In our case, the tip of the defibrillator lead was outside the epicardium. We observed this on CT images and were able to extract the lead without complications.

It is important for clinicians to be aware that delayed right ventricular perforation can result in sudden hemodynamic compromise indicative of cardiac tamponade. Once right ventricular perforation beyond the epicardium is suspected, we should not hesitate to intervene surgically.

## Conflict of Interest

Authors declare no conflict of interest.

## Authorship

MN: performed the CRT‐D implantation. TN: assisted with the CRT‐D implantation. YK: performed the lead extraction surgery and participated in the patient’s hospital care. KS: performed the intrasurgical transesophageal echocardiography. KO: Chief of Cardiology, oversaw the patient's care and treatment plan during CRT‐D implantation. MT: Chief of Cardiac Surgery, assisted with the lead extraction surgery and managed the patient's postoperative care. HW: saw the patient during his follow‐up visits to the pacemaker clinic. MN: drafted the case report, and TN: assisted by polishing the report and revising it in response to the reviewers' comments. All authors have read and approved the final manuscript for publication in *Clinical Case Reports*.

## References

[1] Khan, M. N. , G. Joseph , Y. Khaykin , K. M. Ziada , and B. L. Wilkoff . 2005 Delayed lead perforation: a disturbing trend. Pacing Clin. Electrophysiol. 28:251–253. 15733190 10.1111/j.1540-8159.2005.40003.x

[2] Hirschl, D. A. , V. R. Jain , H. Spindola‐Franco , J. N. Gross , and L. B. Haramati . 2007 Prevalence and characterization of asymptomatic pacemaker and ICD lead perforation on CT. Pacing Clin. Electrophysiol. 30:28–32. 17241311 10.1111/j.1540-8159.2007.00575.x

[3] Migliore, F. , A. Zorzi , E. Bertaglia , L. Leoni , M. Siciliano , M. De Lazzari , et al. 2014 Incidence, management, and prevention of right ventricular perforation by pacemaker and implantable cardioverter defibrillator leads. Pacing Clin. Electrophysiol. 37:1602–1609. 25131984 10.1111/pace.12472

[4] Migliore, F. , L. Leoni , G. Torregrossa , C. Guglielmi , G. Tarantini , G. Buja , et al. 2010 Asymptomatic right ventricular perforation by an implantable cardioverter defibrillator lead detected by home monitoring system. J. Electrocardiol. 43:673–675. 20888001 10.1016/j.jelectrocard.2010.07.022

[5] Mahapatra, S. , K. A. Bybee , T. J. Bunch , R. E. Espinosa , L. J. Sinak , M. D. McGoon , et al. 2005 Incidence and predictors of cardiac perforation after permanent pacemaker placement. Heart Rhythm 2:907–911. 16171740 10.1016/j.hrthm.2005.06.011

[6] Laborderie, J. , L. Barandon , S. Ploux , A. Deplagne , B. Mokrani , S. Reuter , et al. 2008 Management of subacute and delayed right ventricular perforation with a pacing or an implantable cardioverter‐defibrillator lead. Am. J. Cardiol. 102:1352–1355. 18993154 10.1016/j.amjcard.2008.07.025

[7] Krivan, L. , M. Kozák , J. Vlasínová , and M. Sepsi . 2008 Right ventricular perforation with an ICD defibrillation lead managed by surgical revision and epicardial leads–case reports. Pacing Clin. Electrophysiol. 31:3–6. 18181900 10.1111/j.1540-8159.2007.00917.x

